# De novo, heterozygous, loss‐of‐function mutations in *SYNGAP1* cause a syndromic form of intellectual disability

**DOI:** 10.1002/ajmg.a.37189

**Published:** 2015-06-15

**Authors:** Michael J. Parker, Alan E. Fryer, Deborah J. Shears, Katherine L. Lachlan, Shane A. McKee, Alex C. Magee, Shehla Mohammed, Pradeep C. Vasudevan, Soo‐Mi Park, Valérie Benoit, Damien Lederer, Isabelle Maystadt, DDD study, David R. FitzPatrick

**Affiliations:** ^1^ Sheffield Children's Hospital NHS Foundation TrustWestern BankSheffieldUK; ^2^ Clinical Genetics DepartmentAlder Hey Children's NHS Foundation TrustLiverpoolUK; ^3^ Department of Clinical Genetics, Churchill HospitalOxford University Hospitals NHS TrustOxfordUK; ^4^ Wessex Clinical Genetics ServiceUniversity Hospitals SouthamptonSouthamptonUK; ^5^ Human Genetics and Genomic MedicineFaculty of Medicine, University of SouthamptonSouthamptonUK; ^6^ Department of Genetic MedicineBelfast City HospitalBelfastUK; ^7^ Department of Clinical GeneticsGuy's and St. Thomas' Hospital NHS TrustLondonUK; ^8^ Department of Clinical Genetics, University Hospitals of Leicester NHS TrustLeicester Royal InfirmaryLeicesterUK; ^9^ East Anglian Medical Genetics Service, Clinical GeneticsCambridge University Hospitals NHS Foundation TrustCambridgeUK; ^10^ Centre de Génétique HumaineInstitut de Pathologie et de Génétique (I.P.G.)Gosselies (Charleroi)Belgium; ^11^ DDD StudyWellcome Trust Sanger InstituteHinxton, CambridgeUK; ^12^ MRC Human Genetics Unit, Institute of Genetics and Molecular Medicine (I.G.M.M.)University of EdinburghUK

**Keywords:** *SYNGAP1*, 6p21.3 microdeletion, intellectual disability, epilepsy, syndrome, hypertrichosis, strabismus, hip dysplasia, DDD study, behavioral phenotype

## Abstract

De novo mutations (DNM) in *SYNGAP1*, encoding Ras/Rap GTPase‐activating protein SynGAP, have been reported in individuals with nonsyndromic intellectual disability (ID). We identified 10 previously unreported individuals with *SYNGAP1* DNM; seven via the Deciphering Developmental Disorders (DDD) Study, one through clinical analysis for copy number variation and the remaining two (monozygotic twins) via a research multi‐gene panel analysis. Seven of the nine heterozygous mutations are likely to result in loss‐of‐function (3 nonsense; 3 frameshift; 1 whole gene deletion). The remaining two mutations, one of which affected the monozygotic twins, were missense variants. Each individual carrying a DNM in *SYNGAP1* had moderate‐to‐severe ID and 7/10 had epilepsy; typically myoclonic seizures, absences or drop attacks. 8/10 had hypotonia, 5/10 had significant constipation, 7/10 had wide‐based/unsteady gait, 3/10 had strabismus, and 2/10 had significant hip dysplasia. A proportion of the affected individuals had a similar, myopathic facial appearance, with broad nasal bridge, relatively long nose and full lower lip vermilion. A distinctive behavioral phenotype was also observed with aggressive/challenging behavior and significant sleep problems being common. 7/10 individuals had MR imaging of the brain each of which was reported as normal. The clinical features of the individuals reported here show significant overlap with those associated with 6p21.3 microdeletions, confirming that haploinsufficiency for *SYNGAP1* is responsible for both disorders. © 2015 The Authors. *American Journal of Medical Genetics Part A* Published by Wiley Periodicals, Inc.

## INTRODUCTION

De novo mutations are an important cause of moderate and severe intellectual disability (ID). Heterozygous, de novo loss‐of‐function mutations in *SYNGAP1* have been described in 26 individuals to date [Hamdan et al., 2009, 2011a, b; Krepischi et al., 2010; Pinto et al., 2010; Vissers et al., 2010; Zollino et al., 2011; de Ligt et al., 2012; Rauch et al., 2012; Berryer et al., 2013; Carvill et al., 2013; Writzl and Knegt, 2013; Redin et al., 2014]. *SYNGAP1* encodes Ras/Rap GTPase‐activating protein SynGAP, which is a major component of the post‐synaptic density that regulates synaptic plasticity and ERK/MAPK signaling probably via N‐methyl‐d‐aspartate (NMDA) receptor activation [Komiyama et al., 2002; Muhia et al., 2010]. *SYNGAP1* [603384] has been coded in Online Mendelian Inheritance in Man (OMIM^®^) as causing mental retardation, autosomal dominant 5 [612621].

In 2009, Hamdan et al. first reported the sequencing of *SYNGAP1* in 94 apparently nonsyndromic individuals with intellectual disability; they found de novo mutations in three, thus first‐describing this gene as a cause of nonsyndromic intellectual disability (ID) in humans [Hamdan et al., 2009]. This group subsequently published eight further affected individuals through re‐sequencing predominantly ID cohorts enriched for epilepsy [2011a, b; Berryer et al., 2013]. Carvill et al. performed massively parallel sequencing in 500 individuals with epileptic encephalopathy and identified four patients with de novo *SYNGAP1* mutations [Carvill et al., 2013]. Further patients have been described as part of large next generation sequencing studies of individuals with ID [Vissers et al., 2010; de Ligt et al., 2012; Rauch et al., 2012; Redin et al., 2014].

In addition, there have been four individuals with genomic deletions of 6p23.1 involving *SYNGAP1*, and one with a de novo apparently balanced reciprocal translocation in which one of the breakpoints disrupts *SYNGAP1* [Krepischi et al., 2010; Pinto et al., 2010; Klitten et al., 2011; Zollino et al., 2011; Writzl and Knegt, 2013]. Thus the 26 individuals reported to date consist of 21 intragenic mutations, four whole gene deletions, and one translocation. To date, facial images have only been published in six individuals: three in the seminal Hamdan et al. paper, plus three single patients in subsequent papers [Hamdan et al., 2009; Zollino et al., 2011; Rauch et al., 2012; Writzl and Knegt, 2013].

Here, we present molecular and clinical information on 10 previously unreported individuals with de novo mutations in *SYNGAP1*, most of whom were diagnosed using trio exome sequencing of individuals with undiagnosed developmental disorders. The relatively consistent pattern of clinical features and behavioral anomalies observed in these individuals and in previously reported individuals suggests that there is an emerging *SYNGAP1*‐associated syndrome.

## METHODS

### Patient Ascertainment

Seven of the 10 affected individuals were recruited via UK NHS Regional Genetics Services to the Deciphering Developmental Disorders (DDD) study (www.ddduk.org). The eighth individual (7; Table I) was identified as part of routine investigation of ID via a UK NHS paediatric genetics clinic. These eight individuals were seen by the same Paediatric Geneticist (MJP) in addition to the referring Clinical Geneticists. The ninth and tenth individuals are monozygotic twins who were referred for genetic evaluation to the local multi‐disciplinary clinic for children with intellectual disability. See Table I for a summary of the clinical and molecular findings. The Supporting Information online provides additional clinical details.

**Table I:** Clinical Features of Ten Previously‐Unreported Patients With *SYNGAP1* Haploinsufficiency Reported Herein

Category/Individual	1	2	3	4	5	6	7	8	9	10
DECIPHER ID	259041	259840	258913	264135	259214	259606	258536	258536	LEM300469	LEM300468
Mutation details										
Genomic coordninates (chr6; hg19)	chr6 g.33406569 CTGTATG>CTG	chr6 g.33400583G>A	chr6 g.33411111C>T	chr6 g.33411093C>T	chr6 g.33400498 AAACGAACGAA> AAACGAA	chr6 g.33411102CT>C	deletion chr6:33201710‐ 33595089	chr6 g.33411606C>T	chr6 g.33405662T>C	
VEP prediction	Transcript; ENST00000418600 frameshift_variant p.LYE517‐519LX	Transcript; ENST00000418600 missense_variant p.R170Q	Transcript; ENST00000418600 stop_gained p.Q928*	Transcript; ENST00000418600 stop_gained p.R922*	Transcript: ENST00000418600 frameshift_variant p.KRTK142‐145KRX	Transcript; ENST00000418600 frameshift_variant p.L925X	multi‐gene deletion; SYNGAP1 plus 18 others	Transcript; ENST00000449372 stop gained p.Q1079*	Transcript; NM_006772.2 missense_variant p.Leu327Pro	
Inheritance	de novo	de novo	de novo	de novo	de novo	de novo	de novo	de novo	de novo	de novo
Age (years)	7	8	7	3	8	12	5	8	14	14
Sex	Female	Female	Female	Female	Male	Female	Female	Female	Male	Male
Prenatal growth										
Gestation (weeks)	40	30	40	41	40	40	41	40	35	35
Birth weight (g) [z score]	4100 [1.67]	1360 [0.07]	3090 [−0.68]	3600 [0.2]	3460 [0.21]	3180 [−0.46]	3190 [−0.78]	3650 [0.65]	2465 [−0.11]	2460 [−0.12]
Head circumference at birth (cm) [z score]	−	−	−	−	−	−	−	−	32 [−1]	31.5 [−1.3]
NICU admission	No	4 weeks	No	No	No	No	No	No	−	−
Postnatal growth										
Age when measured (yrs)	7.1	8.0	7.1	3.1	8.1	12.1	5.1	8.1	8 y 3 mo	8 y 3 mo
Height (cm) [z score]	117.5 [−0.82]	132 [0.85]	116.4 [−1.03]	93.5 [−.0.45]	120 [−1.5]	131.6 [−2.6]	103 [−1.4]	132.5 [0.85]	119 [−1.8]	110 [−3.4]
Weight (kg) [z score]	24.8 [0.42]	37.7 [2.0]	22.7 [−0.15]	11.5 [−1.98]	23.5 [−0.69]	29.4 [−1.86]	17.2 [−0.55]	30.5 [0.89]	23 [−0.98]	16 [−4.4]
Head circumference (cm) [z score]	50.7 [−1.6]	54 [0.81]	49.5 [−2.6]	47.4 [−2.5]	52.2 [−1.1]	52 [−1.83]	48.2 [−2.9]	53 [−0.03]	52.4 [−0.98]	52 [−1.2]
Facial dysmorphology	Long nose; broad ‐sal bridge; full lower lip	Broad ‐sal bridge; full lower lip	Long nose; broad ‐sal bridge; small ears; full lower lip	Long nose; broad ‐sal bridge; full lower lip	Triangular face; protuberant ears	Long nose; full lower lip	Long nose; broad ‐sal bridge; small ears; full lower lip	Broad ‐sal bridge; full lower lip; ptosis	Deep‐set eyes, high ‐sal bridge; long columella; high‐arched palate	Deep‐set eyes, high ‐sal bridge; long columella; high‐arched palate
Neurology, behavior, development										
Intellectual disability	Moderate	Moderate	Moderate	Moderate	Moderate	Moderate	Moderate	Moderate	Severe	Severe
Sat unaided (months)	20	7	12	24	15	Uncertain	7	12	24–36	24–36
Walked unaided (months)	24	36	60	NYA	17	24	19	30	>60	>60
Speech	40–50 single words; occasional two‐word sentences	Several single words; signs	Occasional single word	NYA	∼200 single words	20 single words; Points; Signs	Two‐word sentences	Four‐word sentences; echolalia	NYA	NYA
Behavior	Aggressive (self & others); routine‐orientated	Aggressive (others); routine‐orientated; obsessions	Aggressive (self & others); routine‐orientated; hand stereotypies	Aggressive (self & others); obsessions (water)	Aggressive (self); obsessions (doors)	Aggressive (others); hand flapping when excited	Aggressive (self & others); obsessions (water)	Autistic spectrum disorder	Routine‐orientated, fasci‐tion with water; laughter outbursts	Routine‐orientated, fasci‐tion with water; laughter outbursts
Autism	−	Yes	No	Yes	Yes	Yes	−	No	Yes	Yes
Sleep disturbance	Yes	Yes	Yes	Yes	Yes	Yes	Yes	Yes (not severe)	Yes	Yes
Hypotonia	No	Yes	Yes	Yes	Yes	No	Yes	Yes	Yes	Yes
Seizures	No	Yes	Yes	Yes	No	Yes	No	Yes	Yes	Yes
Seizure age‐of‐onset	−	6 y	2 y	2 y	−	3 y	−	5 y	13 m	13 m
Seizure type	−	Myoclonic; absences	Myoclonic; absences; drop attacks	Absences; drop attacks	−	Head drops & blinking, visually triggered by patterns	−	Absences (possible); drop attacks	Febrile; absences; drop attacks; occasional tonic‐clonic & myoclonic	Febrile; absences; drop attacks; occasional tonic‐clonic & myoclonic
Gait	Unsteady gait	Wide‐based gait	Wide‐based gait	−	−	Wide‐based gait	Unsteady gait	−	Ataxic	Ataxic
Brain MRI	ND	Normal	Normal	Normal	Normal	Normal	ND	ND	Normal	Normal
Skeletal issues	No	Hip dysplasia (unilateral, requiring osteotomy); 5th finger clinodactyly	Pes planus	Pectus excavatum; kyphosis; hip dysplasia (unilateral, requiring open reduction)	Pes planus	No	No	Lordosis	Kyphoscoliosis	Kyphoscoliosis
Other issues	Gastro‐esophageal reflux	Constipation; café‐au‐lait patch (arm)	Constipation; unilateral divergent strabismus	Constipation; gastrostomy planned; hirsutism	Café‐au‐lait patch (intercostal)	Constipation; hirsutism	Hirsutism; unilateral divergent strabismus	Constipation; strabismus (repaired)	Progressive lower limb spasticity	Progressive lower limb spasticity; gastrostomy

Key: NYA, not yet achieved; ND, not done.

### Mutation Analysis

For the seven individuals identified via the DDD study, trio‐based exome sequencing was performed on the affected individual and their parents, as previously described [Wright et al., 2014]. Each affected individual has also had a high‐resolution analysis for copy number abnormalities using array‐based comparative genomic hybridization (aCGH). Putative de novo mutations were identified from exome data using DeNovoGear software [Ramu et al., 2013] and were validated using targeted Sanger sequencing.

The eighth individual (7; Table I) was identified as having a ∼0.39Mb deletion of 6p21.32p21.31, via a service ISCA 8 × 60K BlueGnome Array. The ninth and tenth individuals are monozygous twins from Belgium, who were identified through a local multi‐gene panel and were validated using targeted Sanger sequencing.

## RESULTS

### *SYNGAP1* Mutations

The validated de novo mutations are detailed in Table I. There were 10 individuals, but two are monozygotic twins, so we describe eight mutations and one deletion. Three individuals had nonsense mutations; three had frameshift mutations resulting in early stop codons. One individual (2; Table I) had a missense mutation c.509G>A (ENST00000418600); p.Arg170Gln (ENSP00000403636.2). On SIFT analysis; this was labeled “Deleterious” with a score of 0.01 and on PolyPhen analysis “Possibly damaging” with a score of 0.529. This mutated residue lies within the PH domain (Prosite PS50003) of SynGAP. The monozygotic twins (9 and 10; Table I) had a missense mutation c.1081T>C (ENST00000418600); p.Leu327Pro (ENSP00000403636). The SIFT score is 0, “Deleterious”, and the PolyPhen is 0.983, “Probably Damaging”. This mutation lies within the C2 domain, which is required for RapGAP activity. One individual (7; Table I) had a 0.39 Mb genomic deletion, which encompassed the entire *SYNGAP1* gene and 18 other genes.

### Growth

Birth weight was normal (z score between −2 and 2) for all of the affected individuals. Postnatal growth was normal in five individuals (1, 2, 5, 8 and 9; Table I). Three of 10 had mild microcephaly (z score between −2.5 and −3), with one of these also having weight on a similar centile (4; Table I). Two individuals had significant short stature (Patients 6 and 10; Table I), one of whom was also significantly underweight (Patient 10; Table I).

### Development

Global delay in developmental milestones was present in all patients with an unusual temporal sequence seen in some patients. For example, Patient 5 (Table I) did not sit unaided until 15 months, but walked unaided at 17 months. Typically independent walking was achieved in the third year of life with the subsequent gait being wide‐based and unsteady. Language acquisition was highly variable within this group. Expressive language was delayed with most children using a limited vocabulary of single words. None of the affected individuals were toilet‐trained at the time of assessment.

### Behavior

Seven of the ten individuals showed general hyperexcitability and aggressive behavior, often directed towards others. A disturbed sleep pattern was reported in all patients, with almost all being treated with, or having had a therapeutic trial of, Melatonin. Anecdotally, families reported a high pain threshold and hyperacusis in some affected individuals.

### Neurology

Seven of the 10 individuals had seizures, most commonly complex and generalized, including myoclonic, drop attacks, and absences. Congenital central hypotonia was common. Most of the affected individuals required ankle splints and/or Piedro boots to aid with walking. Seven individuals have had MR imaging of their brains and in each patient this was reported as normal.

### Facial Features

**Figure 1:**
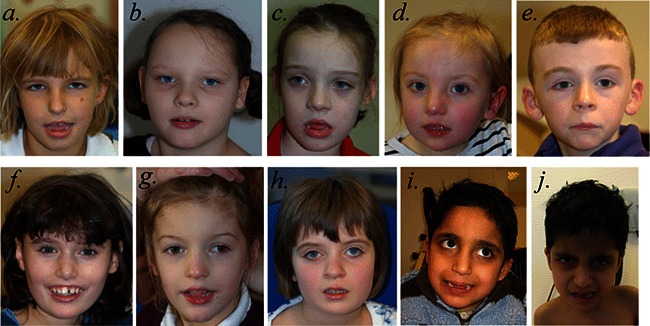
Faces of individuals with *SYNGAP1* haploinsufficiency. Facial photographs of Patient 1 at 7 years, 3 months (**a**); Patient 2 at 8 years, 2 months (**b**); Patient 3 at 7 years, 9 months (**c**); Patient 4 at 3 years, 2 months (**d**); Patient 5 at 8 years, 4 months (**e**); Patient 6 at 12 years, 10 months (**f**); Patient 7 at 5 years, 7 months (**g**); Patient 8 at 8 years, 7 months (**h**); and Patients 9 and 10 at 8 years, 3 months (**i** and **j**). The most common shared facial characteristics are almond‐shaped palpebral fissures, which slant downwards slightly. With the exception of Patient 5 (**e**), the others have a mildly myopathic appearance, with an open mouth and relatively full lower lip. Patients 1 (**a**), 3 (**c**), 4 (**d**), 6 (**f**), 7 (**g**) and 9 (**i**) and 10 (**j**) have relatively long noses; Patients 2 (**b**), 4 (**d**), 5 (**e**), 6 (**f**), 7 (**g**) and 8 (**h**) have relatively long ears with protuberant lobes. Patients 1 (**a**), 6 (**f**) and 9 (**i**) and 10 (**j**) were thought to have relatively deep‐set eyes and Patient 8 (**h**) has a degree of ptosis. Patient 6 (**f**) has a missing central incisor due to trauma. We do not believe that Patient 7 (**g**), the only deletion patient in this series, differs significantly in appearance from the others.

The facial appearance of the ten affected individuals is shown in Figure 1. The most common shared facial characteristics are almond‐shaped palpebral fissures, which slant downwards slightly. All but one had a mildly myopathic appearance. An open‐mouthed appearance, with a relatively full lower lip vermilion was common, as was a low‐hanging columella. 7/10 had relatively long noses (sometimes with under‐development of the ala nasi); 6/10 had relatively long ears with protuberant lobes; 4/10 had relatively deep‐set eyes; and one had a degree of ptosis. There was no obvious difference in facial appearance between the deletion and intragenic mutation patients.

### Other Features

Five of the individuals had constipation, requiring medical therapies; three individuals had fine hirsutism, especially noticeable over limbs and spine; two had significant hip dysplasia, requiring surgical management; three had a kyphosis or kyphoscoliosis; and one had a pectus excavatum (Table I).

## DISCUSSION

*SYNGAP1* was originally reported as causing non‐syndromal intellectual disability [Hamdan et al., 2009]. Supplementary Table I summarizes the available clinical data on the 26 individuals who have been reported to date with presumed causative mutations in *SYNGAP1* or deletions or translocations involving this gene [Hamdan et al., 2009, 2011a, b; Krepischi et al., 2010; Pinto et al., 2010; Vissers et al., 2010; Cook, 2011; Klitten et al., 2011; Zollino et al., 2011; Clement et al., 2012; de Ligt et al., 2012; Rauch et al., 2012; Berryer et al., 2013; Carvill et al., 2013; Writzl and Knegt, 2013; Dyment et al., 2014; O'Roak et al., 2014; Redin et al., 2014]. De novo mutations in this gene are undoubtedly a significant cause of intellectual disability, accounting for 0.62% of all the patients in the DDD Study [Wright et al., 2014] and major contributors to other cohorts that have been studied (Supplementary Table II).

The original designation of the phenotype associated with *SYNGAP1* haploinsufficiency as non‐syndromal is understandable given the generally normal antenatal growth parameters and the relative normality of post‐natal growth. In addition, all patients have a moderate‐to‐severe intellectual disability with few structural anomalies reported on brain imaging. The genomic pathology is also remarkably consistent with almost all patients having heterozygous, de novo, loss of function mutations. The associated genetic mechanism is very likely to be haploinsufficiency given the similarity of the intragenic mutations with the whole gene deletions.

Although there is wide variability in the type and severity of the clinical features associated with *SYNGAP1* haploinsufficiency, some aspects of the phenotype show a level of consistency that suggests *SYNGAP1* haploinsufficiency may be associated with a clinically recognizable syndrome. The seizure type and the behavioral phenotype were relatively consistent in our cohort. Myoclonic, absence and drop attack seizures are typical, both in the reported individuals and those presented in this paper. General hyperexcitability, sleep disturbance and aggressive behavior, often directed towards others, are common features in our cohort and are mentioned in some of the previously reported patients. Clearly these distressing behavioral components of the phenotype require further investigation. Facial photographs were not available in most of the previous reports, but in the cohort presented here a subtle but consistent facial appearance is suggested, although further observations will be required to determine if this is in any way discriminative. The pattern of growth may also be helpful in making a clinical diagnosis. Six of 18 reported patients with postnatal head circumferences recorded, and 3/10 of the patients reported here, had measurements of two standard deviations below the mean for their age. A mild postnatal microcephaly is clearly over‐represented in this group.

In our cohort, 8/10 patients had previously been investigated for Angelman syndrome. There are some similarities with this condition, although we believe that they are clinically distinguishable. Nevertheless, we believe that *SYNGAP1* should also be added to the expanding list of differential diagnoses for Angelman syndrome or patients presenting with Angelman‐like features.

## CONCLUSION

*SYNGAP1* has previously been described as presenting in a non‐syndromal manner. Mutations in this gene have been found to be a relatively‐common cause of intellectual disability in large‐scale massively parallel sequencing studies, where subjects are usually recruited because a clinical syndromal diagnosis has not previously been made. It is arguable whether the term non‐specific may be more appropriate to many subjects recruited into such studies, who most likely represent a heterogeneous mix of those genuinely non‐syndromal, but also of some syndromes more subtle in their associations and/or dysmorphology. For *SYNGAP1* we consider discriminative features in individuals with moderate‐to‐severe ID to be the characteristic facial features, seizure type and behavioral phenotype (generalized hyper‐excitability, sleep disturbance and a propensity to aggression). It is not yet clear if hypotonia, hip dysplasia, strabismus, wide‐based/unsteady gait, fine hirsutism (limbs and spine), and significant constipation are helpful discriminators. Some patients have microcephaly, but growth parameters are generally within the normal range.

## Supporting information

Additional supporting information may be found in the online version of this article at the publisher's web‐site.

**Supporting Information Table S1**: Summarizes the available clinical data on the 26 individuals who have been previously‐reported with presumed causative mutations in *SYNGAP1*, deletions or translocations involving this gene.

**Supporting Information Table S2**: Summarizes the *SYNGAP1* pick‐up rate in NGS studies in which *SYNGAP1* patients have previously been reported.

Supplementary Materials.
