# Supplementary material for: De novo, heterozygous, loss‐of‐function mutations in SYNGAP1 cause a syndromic form of intellectual disability
Source: Am J Med Genet A. 2015 Jun 15;167(10):2231–7. doi: 10.1002/ajmg.a.37189 (PMC4744742; doi:10.1002/ajmg.a.37189)
Supplement: Supplementary file 2 — Supporting Information Table S2: Summarizes the SYNGAP1 pick‐up rate in NGS studies in which SYNGAP1 patients have previously been reported. [file AJMG-167-2231-s003.doc]

Supplementary Table 2: *SYNGAP1* Pick-up Rates in NGS Studies

| **Study** | **Cohort** | **Patients with pathogenic SYNGAP1 mutations** | **Mutation Yield** |
| --- | --- | --- | --- |
| Hamdan et al. 2009 | NSID | 3/94 (Targeted sequencing) | 3% |
| Vissers et al. 2010 | NSID | 1/10 (Exome sequencing) | 10% |
| Hamdan et al. 2011 | NSID | 3/60 (Targeted sequencing) | 5% |
| Berryer et al. 2013 | NSID (*) | 5/34 (Targeted & Exome sequencing) | 15% |
| De Ligt et al. 2012 | ID | 1/100 (Exome sequencing) | 1% |
| Rauch et al. 2012 | NSID | 2/51 (Exome sequencing) | 4% |
| Carville et al. 2013 | Epileptic-  Encephalopathy | 5/500 (Targeted sequencing) | 1% |
| Redin et al. 2014 | NSID | 1/104 (Targeted sequencing) | <1% |
| DDD (ongoing) | "Severe" phenotypes | 7/1139 (Exome sequencing) | <1% |

* Enriched for cases with epilepsy
