# Supplementary material for: De novo, heterozygous, loss‐of‐function mutations in SYNGAP1 cause a syndromic form of intellectual disability
Source: Am J Med Genet A. 2015 Jun 15;167(10):2231–7. doi: 10.1002/ajmg.a.37189 (PMC4744742; doi:10.1002/ajmg.a.37189)
Supplement: Supplementary file 3 — Supplementary Materials. [file AJMG-167-2231-s001.docx]

**SUPPLEMENTARY MATERIALS**

**Clinical descriptions**

**Patient 1** (a; Table I)

This 7-year-old girl has a healthy, older maternal half-brother. Her parents are healthy and unrelated and there is no significant family history. She was conceived naturally and mother was well during the pregnancy and on no regular medications at that time. She was a full-term, normal vaginal delivery, with a birth-weight of approximately 4.10kg. She did not go to the Special Care Baby Unit. However, she had to be woken for feeds initially and was treated for gastro-esophageal reflux (GOR). She is generally well and has not been diagnosed with a seizure disorder. She first sat unaided aged 20 months and walked independently aged two years. At age of seven years, she had 40-50 single words. Her behavior is a significant problem: she pulls own hair, spits, kicks, and can be aggressive towards her brother in particular. She would seem to struggle particularly with transition/change. On examination, she has the following dysmorphic facial features: relatively long nose, full lower lip. Her growth parameters at the age of seven years, three months are: height approximately 117.5cm (9^th^-25^th^ centiles), weight approximately 24.8kg (50^th^-75^th^ centiles), and OFC approximately 50.7cm (2^nd^-9^th^ centiles). Previous investigations have included: normal Array; normal molecular genetic testing for fragile-X and Angelman syndrome (methylation); no plausible variants detected on DDD Study Agilent custom array-CGH (genomic plus five probes per exon); she has not had an MRI or EEG performed to-date.

**Patient 2** (b; Table I)

This 8-year-old girl is the only child of healthy, unrelated parents; there is no significant family history. She was conceived naturally. Mother experienced bleeding throughout her pregnancy, although no problems were detected on ultrasound scanning. She was delivered by emergency caesarean at 30 weeks gestation, following premature rupture of membranes. Her birth-weight was approximately 1.36kg. She spent her first four weeks on the Special Care Baby Unit, with a relatively uneventful course, requiring naso-gastric tube-feeding and phototherapy for jaundice only. She was feeding well on discharge. Congenital dysplasia of the left hip was diagnosed at the age of nine months and this eventually required an osteotomy of her left hip, aged approximately three years. She also had an abscess drained from the back of her neck aged two years. She is otherwise generally well. She developed myoclonic absence seizures from the age of six years and these are partly-controlled by Sodium Valproate. She has low muscle tone and requires regular laxatives for constipation. She has no problems with her hearing and vision. Her parents report her to have a relatively-high pain threshold and she does not cry often. She wears Piedro boots and has a wide-based gait, and also struggles to walk on gradients. She also struggles to hold a pen. She first sat independently aged seven months and walked independently aged three years (not having previously crawled). She was delayed in speech acquisition and now uses several, largely single words. Her behavior can be challenging: biting, scratching and kicking others. This can be exacerbated by a change in her routine. She prefers to eat with her fingers and has a limited diet (largely potatoes). She is not yet fully toilet-trained. She has been labelled with an autistic spectrum disorder. On examination, she has the following dysmorphic facial features: relatively-broad nasal bridge and full lower lip vermilion. She has mild fifth-finger clinodactyly and one very small cafe-au-lait patch on her arm. Her growth parameters at the age of eight years, two months were: height approximately 132.0cm (75^th^-91^st^ centiles), weight approximately 37.7kg (98^th^ - 99.6^th^ centiles), and ofc approximately 54.0cm (75^th^-91^st^ centiles). Previous investigations have included: normal molecular genetic analysis for Angelman syndrome (methylation), myotonic dystrophy, fragile-X and Rett syndrome (*MECP2*); no plausible variants detected on DDD Study Agilent custom array-CGH (genomic plus five probes per exon); MRI brain normal; EEG - generalised-onset of rhythmic and regular spike and slow waves discharges and also polyspike activity lasting for 2 to 5 seconds associated clinically with a myoclonic absence; inter-ictal recording showed a diffusely slow and other generalised spike and slow wave discharges.

**Patient 3** (c; Table I)

This 7-year-old girl has a younger sister and two older paternal half-brothers. Her parents are healthy and unrelated. They previously suffered a miscarriage due to Listeria infection, but there is no other significant family history. She was conceived naturally and mother was well during the pregnancy and taking no regular medications at that time. There were no problems anticipated on antenatal ultrasound screening. She was a full-term normal delivery with a birth-weight of the approximately 3.09kg. She did not go to the Special Care Baby Unit and had no feeding problems. The parents first noted developmental delay aged approximately nine months, when she was not sitting independently. She also has low muscle tone. She was diagnosed with seizures aged two years; these take the form of brief myoclonic episodes with head dropping and she currently takes Sodium Valproate and Lamotrogine. She also takes regular laxatives and Melatonin for sleep disturbance. She is prone to upper respiratory tract infections, but is otherwise generally well. She first sat unsupported aged approximately one year and walked independently from the age of five years. She has insoles in her shoes and has a wide-based gait. She screams when excited and uses occasional single words. She “eye points” and physically leads parents to what she wants. She failed the initial neonatal hearing test and subsequently passed hearing tests. She has no visual problems. She is not yet toilet-trained. She still drinks lots of milk, can eat pureed and some finger foods, but has an aversion to spoons. She is said to be very loving and have a good sense of humor. However, she can have challenging behavior and “transition issues”. These are usually provoked by change of routine and/or environment. She can bite hands, cling to chairs, bite others, pinch and grab. She likes sensory things, such as lights and spinning, but she has no particular obsessions. On examination, she has the following dysmorphic facial features: long nose, with relatively-broad nasal bridge; small ears; full lower lip vermilion. She also has pes planus, a wide-based gait and wears insoles. She has a right divergent strabismus (exotropia). Her growth parameters at the age of seven years, nine months are: height approximately 116.4cm (2^nd^-9^th^ centiles), weight approximately 22.7kg (25^th^-50^th^ centiles), and ofc approximately 49.5cm (< 0.4^th^ centiles). Previous investigations have included: paternally-inherited 460kb duplication of 6q26 on 180k oligoarray; normal molecular genetic testing for Fragile-X, Angelman syndrome (methylation and *UBE3A*) and Rett syndrome (*MECP2* and *CDKL5*); no plausible variants detected on DDD Study Agilent custom array-CGH (genomic plus five probes per exon); MRI - normal; EEG - high-amplitude slow activity; frequent bursts of polyspike and wave activity; anterior dominant slow spike and wave at 1-2Hz.

**Patient 4** (d; Table I)

This 3-year-old girl is the middle child of three born to healthy, unrelated parents; there is no significant family history. She was conceived naturally and mother was well during pregnancy and taking no regular medications at that time. There were no problems anticipated on antenatal ultrasound screening. She was a post-term, normal vaginal delivery with a birthweight of approximately 3.60kg. She did not go to the Special Care Baby Unit. Her mother first thought she may have some problems around the age of 4-5 months, as her eyes did not appear to be focusing. She was referred to an Ophthalmologist and subsequently onto Pediatric services. She was reported to be a quiet baby for the first year. She bottle-fed well initially, but then struggled to wean. She was diagnosed with a dysplasia of her right hip coincidentally following an MRI scan of her brain and spine aged one year. This has been treated by an open reduction operation. She has significant hypotonia and has developed a kyphosis secondary to this. She uses Piedro boots and a standing frame. She is prone to cold extremities, especially her feet. She was first noted to have drop seizures aged two years and also has absence seizures. These are currently well-controlled on Sodium Valproate. She also takes regular laxatives and Melatonin for a disturbed sleep pattern. She is not yet toilet-trained. She has glasses for long-sightedness, though does not tolerate wearing these; she also has nystagmus, especially when tired. She first sat independently from the age of around two years. She rolls rather than crawling and cannot yet walk independently. She screams when excited, but she has no discernible words yet. Her behavior is challenging: biting, scratching and pulling hair (herself and others). She is said to like new experiences and new people. She struggled with weaning and still has to have pureed food. A gastrostomy is planned. She attends a special school. She is said to be very “sensory” and has an obsession with water. On examination, she has the following dysmorphic facial features: long nose, with relatively broad nasal bridge; full lower lip vermilion. She has a significant pectus excavatum and kyphosis, with some fine hirsutism, especially down her spine. She has significant hypotonia. Her growth parameters at the age of three years, two months are: height approximately 93.5cm (25^th^-50^th^ centiles), weight approximately 11.5kg (0.4^th^-2^nd^ centiles), and ofc approximately 47.4cm (0.4^th^-2^nd^ centiles). Previous investigations have included: normal ISCA 60k oligoarray; normal molecular genetic testing for fragile-X and Angelman syndrome (methylation); no plausible variants detected on DDD Study Agilent custom array-CGH (genomic plus five probes per exon); MRI - ventricular and fronto-temporal subarachnoid space prominence only; EEG - large triangular slow wave activity posteriorly.

**Patient 5** (e; Table I)

This 8-year-old boy is the middle child of three born to healthy, unrelated parents; there is no significant family history. He was conceived naturally and mother was generally well during the pregnancy, but she thought this was a different pregnancy to her others. She had immunoglobulins towards the end of the second trimester, because of Varicella exposure, but there were no abnormalities detected antenatally. He was a post-term normal delivery, with a birth-weight of approximately 3.46kg and he did not go to the Special Care Baby Unit. He had mild, self-limiting jaundice for the first week of life. He struggled to establish feeding and was troubled with gastro-oesophageal reflux (GOR). He had an admission for croup aged approximately two years. He is reported to be particularly prone to respiratory infections, but is otherwise generally well and has had no other hospitalisations. He used to take Melatonin for a poor sleep pattern, but this has now been stopped. His parents report that he used to fall for no obvious reason, but this has been largely attributed to clumsiness (dyspraxia) and he has never formally been diagnosed with a seizure disorder. He has no problems with his vision and hearing. He is prone to feeling cold. He has hypotonia and his parents believe him to have a high pain threshold. He first sat age 13 months and first walked independently between 18 months and two years (never having crawled). His first words came aged two years and he now uses approximately 200 single words. He is reportedly to be generally quiet and not especially affectionate. His behavior can be problematic: he used to head-bang and now can slap own head and can also bite and nip himself. He can hit and pull hair of younger sibling, but not his older sibling or parents. He has no particular issues with routine. He is not yet fully toilet-trained, but is not troubled with constipation. He loves bath times and is obsessed with opening and closing doors. His behavior can also be problematic in crowds. He has a limited diet, especially at home: largely cereals, pasta and potatoes. He can use a spoon and fork, but struggles to use a knife. On examination, he has the following dysmorphic facial features: triangular face; protuberant ears. He has some skin hyperpigmention, possible café-au-lait patch-type, in a left lower intercostal distribution. He has pes planus and everted ankles. He has normal male genitalia. His growth parameters at the age of eight years, four months are: height approximately 120cm (2^nd^-9^th^ centiles), weight approximately 23.5kg (9^th^-25^th^ centiles), and ofc approximately 52.2cm (9^th^-25^th^ centiles). Previous investigations have included: normal karyotype and MLPA; normal molecular genetic testing for Fragile-X; no plausible variants detected on DDD Study Agilent custom array-CGH (genomic plus five probes per exon); no intracranial abnormality was demonstrated on MRI; EEG - not performed.

**Patient 6** (f; Table I)

This 12-year-old girl is the oldest child of healthy, unrelated parents with two healthy younger brothers. There is no significant family history. The mother was well during pregnancy and on no regular medications at that time. She was a full-term normal delivery, weighing 3.18kg. She did not go to the Special Care Baby Unit. She struggled to establish breast-feeding initially. She is generally well and has had no overnight hospitalisations. Seizures first developed at age three years. These take the form of petit mals with nodding and episodes of falling asleep and are now well-controlled on Sodium Valproate. She also takes Risperidone and Melatonin for her behavior and sleep problems, plus regular laxatives. There are no problems with her vision and hearing. She previously suffered the traumatic loss of one of her central incisors. Her development is significantly delayed and she first walked aged approximately two years. At her current age of nearly 13 years, she has 20-30 single words. She points and signs largely for communication. Before commencing Melatonin, she struggled to go to sleep at night, with occasional waking, although she is not a particular early-riser. She uses a fork, but struggles with foods, which are difficult to chew. She is not yet toilet-trained. She is prone to agitation and her behavior can be extremely challenging (biting, scratching and kicking). She has stereotypic movements and hand flaps when excited and drools excessively. She is very “sensory”; staring at stripes can induce seizures. She is also prone to hyperacusis. She has been labelled with an autistic spectrum disorder and has a number of obsessive traits. On examination, she has a relatively long nose, full lower lip vermilion, gaps between her teeth and deep-set eyes. She has tapering fingers and a degree of hirsutism on the arms and legs. She has always had a wide-based, ataxic gait. Her growth parameters at the age of 12 years, 10 months are: height approximately 131.6cm (<0.4^th^ centiles), weight approximately 29.4kg (0.4^th^-2^nd^ centiles), and ofc 52.0cm (2^nd^ -9^th^ centiles). Previous investigations have included: normal service oligonucleotide Array; normal molecular genetic testing for fragile-X, Angelman syndrome (methylation and *UBE3A*), Rett syndrome (*MECP2* and *CDKL5*) and *SCN1A*; no plausible variants detected on DDD Study Agilent custom array-CGH (genomic plus five probes per exon); MRI - normal; EEG - slightly slowed background; spike and polyspike-and-wave discharges bilaterally, most prominent over right temporal region, increasing with visual stimuli.

**Patient 7** (g; Table I)

This 5-year-old girl has a younger brother and older paternal half-brother. Her parents are healthy and unrelated. Her half-brother is currently being assessed for possible Attention Deficit Hyperactivity Disorder (ADHD). Her father was said to have some mild speech delay as a child, but is of normal intelligence. She was conceived naturally and mother was well during the pregnancy and on no regular medications at that time. There was a degree of oligohydramnios in the pregnancy. She was a normal vaginal delivery, at term plus eight days, with a birth-weight of approximately 3.19kg. She did not go to the Special Care Baby Unit and she had no feeding difficulties. She had an admission with a febrile seizure aged 18 months. She has subsequently been reported to have the occasional absence episode and possible myoclonic movements in her sleep, although she has no formal diagnosis of epilepsy at this time. She is prone to ear infections and has had grommets inserted on one occasion. She has a divergent strabismus of her left eye. She is otherwise generally well and on no regular medications. She first sat independently aged seven months and walked independently aged 19 months, but at her current age (of five years) she remains unsteady. At the age of two, she had the use of ten single words; she currently can make sentences of up to 3-4 words, although she usually uses no more than two words together. She has a degree of hypermobility, struggling with fine motor skills and has previously worn Piedro boots. She attends a Special School. She is said to have a short temper, can smack own head, and smack and head-butt others. She is not particularly bound by routines. Her play can be repetitive with certain obsessions, especially water. She is said to have a short attention span, little regard for danger, and her parents have previously thought she may have a high pain threshold. She still mouths toys and is prone to drooling. She has difficulty getting to sleep, wakes early and then will not go back to sleep. She is said to be greedy with food, with a wide diet and can use cutlery. She is not yet fully toilet-trained. On examination, she has the following dysmorphic facial features: small, low set ears; relatively long nose, with wide nasal bridge; full lower lip vermilion. She has a divergent, left-sided strabismus. She has some fine hirsuitism on her limbs and down her spine. She also walks with an unsteady/ataxic gait. Her growth parameters at the age of five years, seven months are: height approximately 103cm (0.4^th^-2^nd^ centiles), weight approximately 17.2kg (9^th^-25^th^ centiles), and ofc 48.2cm (<0.4^th^ centiles). On a service ISCA 8x60K BlueGnome Array, she was identified as having a ~0.39Mb deletion of 6p21.32p21.31, which contained 16 annotated genes, three of which have OMIM Morbid entries (*SYNGAP1*, *TAPBP* and *ITPR3*). This also detected a paternally-inherited 0.5Mb duplication of 4q24. Other investigations have included: normal molecular genetic testing for fragile-X, and Angelman syndrome (methylation); MRI - not performed; EEG did not show any epileptiform discharges and there was no evidence of epilepsy.

**Patient 8** (h; Table I)

This 8-year-old girl has an older sister, who is well; her parents are healthy and unrelated. There is no other significant family history. There was polyhydramnios towards the end of the pregnancy and mother noted reduced fetal movements relative to the previous pregnancy. She was delivered by caesarean section, because of a breech lie, at 39^+5^ weeks gestation. Her birth-weight was approximately 3.65kg and she did not go to the Special Care Baby Unit. However, she was noted to be generally floppy and a relatively quiet baby who fed well. She has previously had an operative repair of a divergent strabismus of her right eye. She is generally hypermobile, with ankle orthotics and an ataxic, “high-stepping” gait. She has not been formally diagnosed with epilepsy, although parents describe a previous cluster of five drop attacks and she is also prone to possible absence episodes. Laxatives for chronic constipation are her only regular medication. She first sat independently aged 12 months and walked independently aged 2½ years. At her current age, of eight years, she can make sentences of up to 3-4 words, but tends to echo speech more than speak spontaneously. She has noticeable joint laxity, poor fine motor skills in particular and is sensitive to certain noises. She has an erratic sleep pattern. Her behavior is not reported to be particularly problematic. She has a good appetite. She is not yet fully toilet-trained. On examination, she has the following dysmorphic facial features: mild myopathic facies with bilateral partial ptosis, slight downslanting palpebral fissures, persistence of right-sided divergent squint, increased lumbar lordosis, three small resolving capillary hemangiomas on the right forearm, bilateral pes planus and a Beighton scale score of 4/9. She also walks with an unsteady/ataxic gait. Her growth parameters at the age of eight years, seven months are: height approximately 132.5cm (50-75^th^ centiles), weight 30.5kg (50-75^th^ centiles), and ofc 53.0cm (25-50^th^ centiles). Previous investigations have included: normal results on the Affymetrix GeneChip 6.0 SNP array; no plausible variants detected on DDD Study Affymetrix custom array-CGH (genomic plus five probes per exon); other investigations have included: normal molecular genetic testing for Fragile-X and Myotonic Dystrophy; normal serum creatine kinase and limited metabolic screen (plasma amino acids, ammonia and lactate).

**Patients 9 and 10** (i and j; Table I)

These 14-year-old male twins were born from nonconsanguineous and healthy parents. There is no significant family history. During pregnancy, the mother received thyroxin substitution because of a thyroidectomy two years earlier for a benign nodule. The twins were born at 35 weeks gestation, by caesarean, for a suspicion of maternal HELPP syndrome. Neonatal parameters were normal (APGAR score was 9 for Twin 1 and 10 for Twin 2 at five minutes), with birthweight, height and head circumference around the 50^th^ centile. Both patients had neonatal hypotonia and developmental delay. They had poor head control and poor visual contact at five months. At the age of 13 months, both developed febrile seizures, followed by absence epilepsy with drop attacks. They were treated with sodium valproate and lamotrigine. Control of epilepsy was initially poor, but improved with increasing therapeutic dosages. Twin 1 developed occasional tonic-clonic and myoclonic seizures after the age of eight years. Despite early physical therapy, development was severely delayed for both twins, with acquisition of the sitting position between two and three years, and acquisition of walking with aids after five years. They developed progressive lower limb spasticity and kyphoscoliosis. Only Twin 2 could walk without aids at the age of seven years. They can grasp an object with the whole hand, but developed no pincer grasp. They acquired no speech. Both have severe-to-profound intellectual disability, with autistic features. They are dependent on routines and rituals, have a fascination for water, and have poor sensibility to pain. However, they try to attract their parents’ attention by crying and appreciate body contact. They usually have happy behavior with unexplained outbursts of laughter. They show no hyperventilation episodes. They have sleep disturbances. Twin 1 has normal growth development, within the lower range, with height and weight following the 3^rd^ centile, and the head circumference at P50. Twin 2 had marked feeding difficulties, with severe gastro-esophageal reflux. He had growth delay with height at -4 SD and weight at -3.5 SD, with preserved head circumference between P25 and P50. Due to persistent feeding difficulties, he had a gastrostomy at the age of 11 years. On clinical examination, aged eight years, the twins had very similar facial features, but no marked dysmorphism. We noted deep-set eyes, high nasal bridge, long columella and high palate. They had kyphoscoliosis and feet deformities, more marked for Twin 1. They have mild tapering fingers. Both showed axial hypotonia, brisk osteo-tendineous reflexes and spasticity of the lower limbs. They had ataxic, wide-based and spastic gaits. They also had marked orofacial hypotonia with sialorrhea. Brain MRI performed at two years was normal for Twin 2 and showed only mild periventricular leucomalacia sequelae for Twin 1. Metabolic investigations were normal (plasma amino-acids, very long chain fatty acids, orotic acid, acylcarnitines, transferrin electrophoresis, thyroid hormones, alpha-fetoprotein, lactate, pyruvate, ammonia; and urine amino-acids, organic acids, purine and pyrimidine metabolites, creatine, guanidinoacetaten etc.). Array-CGH (Agilent ISCA 180K) revealed no chromosomal rearrangement. Methylation profile of the 15q11-q13 region was normal. Sequencing of the *MECP2*, *UBE3A*, *ARX*, *ATRX* and *TCF4* genes failed to identify a causal mutation.
